# Primary care physician knowledge, attitudes, and diagnostic testing practices for norovirus and acute gastroenteritis

**DOI:** 10.1371/journal.pone.0227890

**Published:** 2020-01-14

**Authors:** Cristina V. Cardemil, Sean T. O’Leary, Brenda L. Beaty, Katy Ivey, Megan C. Lindley, Allison Kempe, Lori A. Crane, Laura P. Hurley, Michaela Brtnikova, Aron J. Hall

**Affiliations:** 1 National Center for Immunization and Respiratory Disease, Centers for Disease Control and Prevention, Atlanta, GA, United States; 2 Adult and Child Consortium for Health Outcomes Research and Delivery Science, University of Colorado School of Medicine and Children's Hospital Colorado, Aurora, CO, United States; 3 Department of Pediatrics, University of Colorado Anschutz Medical Campus, Aurora, CO, United States; 4 Department of Community and Behavioral Health, University of Colorado Anschutz Medical Campus, Aurora, CO, United States; 5 Division of General Internal Medicine, Denver Health, Denver, CO, United States; University of Nevada Reno School of Medicine, UNITED STATES

## Abstract

**Background:**

Norovirus is a leading cause of acute gastroenteritis (AGE) across the age spectrum; candidate vaccines are in clinical trials. While norovirus diagnostic testing is increasingly available, stool testing may not be performed routinely, which can hamper surveillance and burden of disease estimates. Additionally, lack of knowledge of the burden of disease may inhibit provider vaccine recommendations, which could affect coverage rates and ultimately the impact of the vaccine. Our objectives were to understand physicians’ stool testing practices in outpatients with AGE, and physician knowledge of norovirus, in order to improve surveillance and prepare for vaccine introduction.

**Methods:**

Internet and mail survey on AGE, norovirus, and future norovirus vaccines conducted January to March 2018 among national networks of primary care pediatricians, family practice and general internal medicine physicians.

**Results:**

The response rate was 59% (820/1383). During peak AGE season, physicians estimated they ordered stool tests for a median of 15% (interquartile range: 5–33%) of their outpatients with AGE. Stool tests were reported as more often available for ova and parasites, *Clostridioides difficile*, and bacterial culture (>95% for all specialties) than for norovirus (6–33% across specialties); even when available, norovirus-specific tests were infrequently ordered. Most providers were unaware that norovirus is a leading cause of AGE across all age groups (Pediatricians 80%, Family Practice 86%, General Internal Medicine 89%) or that alcohol-based hand sanitizers are ineffective against norovirus (Pediatricians 51%, Family Practice 66%, General Internal Medicine 62%). Concerns cited as major barriers to implementing a future norovirus vaccine included if the vaccine is not covered by insurance (General Internal Medicine 64%, Pediatricians 67%, Family Practice 74%) and lack of adequate reimbursement for vaccination (Pediatricians 43%, General Internal Medicine 46%, Family Practice 50%). Factors that providers believed were ‘not at all a barrier’ or ‘minor barrier’ to new vaccine introduction included the belief that “my patients won’t need this vaccine” (General Internal Medicine 78%, Family Practice 86%, Pediatricians 90%) and “my patients already get too many vaccines” (Family Practice 89%, General Internal Medicine 92%, Pediatricians 95%).

**Conclusions:**

Primary care physicians had few concerns regarding future norovirus vaccine introduction, but have knowledge gaps on norovirus prevalence and hand hygiene for prevention. Also, physicians infrequently order stool tests for outpatients with AGE, which limits surveillance estimates that rely on physician-ordered stool diagnostics. Closing physician knowledge gaps on norovirus burden and transmission can help support norovirus vaccine introduction.

## Introduction

Norovirus is responsible for 20 million illnesses annually in the United States, including approximately 2 million outpatient visits, and is a leading cause of acute gastroenteritis (AGE) across all age groups [1]. The burden of disease is substantial; children <5 years of age have the highest rates of norovirus-associated medical visits[2], while older adults are at greatest risk for norovirus-associated deaths[3, 4]. Several candidate vaccines are currently in clinical trials, including an oral tablet vaccine that recently reported phase I results from a bivalent GI.1/GII.4 formulation in adults, as well as a bivalent GI.1/GII.4 consensus sequence virus-like particle intramuscular vaccine candidate that has conducted phase II trials in children, adults, and the elderly [5–7].

Despite the large burden of disease, practicing physicians may not be aware of the importance of norovirus as a cause of AGE. This is likely due to several factors, including the perception of the disease as mild and self-limiting, as well as the recent shift in the relative importance of norovirus following the successful implementation of the rotavirus vaccine program over the past decade, with norovirus emerging as the leading cause of medically-attended AGE in U.S. children.[3] Additionally, the ability of physicians to diagnose norovirus has historically been hampered by the unavailability of rapid, sensitive assays in clinical settings[8]. In the absence of rapid point of care assays, testing may not always be warranted, such as with patients presenting with mild to moderate illness requiring only supportive care. The interpretation of sensitive PCR-based assays can also be challenging, as multi-pathogen panels may detect organisms that are not necessarily causing disease at the time of testing. However, lack of clinical diagnostic testing greatly impacts public health surveillance efforts, which often rely on physician-ordered testing, thereby limiting development of robust disease burden estimates without implementation of resource-intensive procedures[9].

With the recent expansion of polymerase chain reaction (PCR)-based multi-pathogen diagnostic assays in clinical settings, the ability to detect norovirus has increased rapidly in clinical and research settings[10]. In this study, we aimed to understand physician use of stool diagnostic testing platforms and assess physician knowledge of norovirus and perceptions of future vaccines. Our goal was not to change physician testing practices, but rather to understand the pre-vaccine licensure knowledge and testing practices of physicians who manage AGE in the outpatient setting at a time when diagnostic testing options are expanding. Ultimately, this information will help guide educational campaigns for physicians and patients in preparation for the introduction of future norovirus vaccines. In addition, the results from this survey will inform methods for ongoing surveillance for norovirus and AGE, which can help epidemiologists to more accurately estimate the burden of disease and the impact of future vaccines.

## Methods

### Study population

We utilized national networks of primary care pediatricians (Peds), family practice (FP), and general internal medicine (GIM) physicians who identified as members of national physician organizations (American Academy of Pediatrics [AAP], American Academy of Family Physicians [AAFP], and American College of Physicians [ACP], respectively). Networks were recruited previously and agreed to respond to several surveys each year. We previously demonstrated that recruited physicians were similar to their national membership with respect to provider region, practice location, and setting[11].

### Survey development & administration

We developed a survey to determine physician use of diagnostic testing platforms for norovirus and other AGE pathogens, patient indications for pursuing such testing, knowledge of norovirus, and perceptions of future norovirus vaccine use. At the beginning of the survey, we defined AGE as the presence of vomiting or diarrhea lasting less than two weeks. Physicians were asked to estimate the monthly number of patients seen for any reason, for AGE, and for whom stool tests were ordered, stratified by peak norovirus season (November-April) versus non-peak season (May-October).

We used Likert scales to record physicians’ perception of factors associated with likelihood of stool diagnostic testing in patients with AGE, norovirus severity of illness, agreement on need for diagnostic testing for norovirus, and perceptions of barriers to using future norovirus vaccines. Knowledge of norovirus was assessed through a series of true or false questions.

The survey was pre-tested in a national advisory committee with representation from AAP, AAFP and ACP. Following pre-testing, the survey was piloted among 25 Peds, 5 FP and 18 GIM providers. The final survey was administered from January through March 2018 by internet or mail, based on physician preference, with up to eight email or two mailed reminders. The mail protocol followed Dillman’s tailored design method[12]. The Institutional Review Board at the University of Colorado Denver deemed this study exempt research not requiring written informed consent.

### Statistical analysis

Internet and mail surveys were pooled as prior research indicated the responses attained by either method are similar[12–14]. The monthly number of patients presenting with AGE reported by each respondent was divided by the total monthly number of patients seen to estimate a monthly percentage of patients seen with AGE. The monthly number of patients with stool tests ordered was similarly divided by the monthly number of patients seen with AGE to estimate the percentage of patients with AGE for whom a stool test was ordered. For stool diagnostic tests analysis, responses were grouped under ordered (Always, Often, and Sometimes) or not ordered (Rarely and Never). Item non-response was <5% unless otherwise noted, and missing data were excluded from analyses. Groups were compared using Wilcoxon, chi-square and Fisher’s exact tests. Ranges are reported unless there were significant differences in responses by specialty.

## Results

Overall response rate was 59% (820/1,383) (Table 1). Peds had the highest response rate (68%, 319/466), followed by FP (58%, 266/461) and GIM (52%, 235/456).

**Table 1:** Comparison of respondents and Non-respondents, by specialty (n = 1,383).

			Pediatrics			Family Medicine			General Internal Medicine		
			Respondents (n = 319)	Non-respondents (n = 147)	p-value	Respondents (n = 266)	Non-respondents (n = 195)	p-value	Respondents (n = 235)	Non-respondents (n = 221)	p-value
Gender, %					0.73			0.14			0.33
		Female	35	37		43	36		45	40	
		Male	65	63		57	64		55	60	
Setting, %					0.65			0.14			**0.03**
	Private practice		79	80		69	77		65	75	
	Hospital or clinic		18	15		23	18		27	17	
	HMO		3	5		8	5		8	8	
Location, %											0.99**
		Urban	52	56	0.43**	34	38	0.54	55	55	
		Suburban	46	44		58	55		43	44	
		Rural	1	0		8	7		1	1	
Region, %					0.36						0.62
	Midwest		23	18		31	27	**0.02**	24	19	
	Northeast		22	18		18	13		23	23	
	South		35	41		27	41		31	35	
	West		29	23		24	19		21	23	
Age, years, mean (sd)/ median			50.2 (10.5) / 50.0	52.2 (10.9) / 51.0	0.06	55.2 (8.2) / 55.0	56.0 (7.5) / 57.0	0.30	55.1 (9.1) / 57.0	56.1 (8.8) / 57.0	0.25
Number of providers in practice, mean (sd) / median			11.7 (27.8) / 6.0	11.4 (41.7) / 5.0	0.15*	11.5 (34.6) / 6.0	8.3 (11.5) / 4.0	0.03*	19.4 (44.3) / 7.0	53.4 (491) / 5.0	0.01*

*Wilcoxon test

**Fisher’s Exact test

### Healthcare visits and stool tests ordered for AGE

Pediatric providers reported seeing a greater proportion of their patients for AGE than adult providers, in both peak and non-peak seasons (Peak season: Peds 9%, FP 5%, GIM 3%, p<0.01; non-peak season: Peds 5%, FP 3%, GIM 2%, p<0.01) (Table 2). Overall, physicians reported infrequently ordering stool diagnostics for their patients with AGE (peak 15%, non-peak 20%; overall 17%). By specialty, GIM and FP providers were more likely to order stool tests than Peds in both peak and non-peak seasons (peak: GIM 33%, FP 22%, Peds 10%, p<0.01, non-peak: GIM 33%, FP 29%, Peds 12%, p<0.01), but the median number of patients with stool tests ordered overall was low (2 per month for all 3 specialties in peak season) (Table 3). Notably, 13%-15% of Peds and FP and 53% of GIM were unable to estimate numbers of AGE patients seen and tests ordered and were excluded from the above analyses.

**Table 2:** Monthly healthcare visits for AGE, by specialty and season.

	Pediatrics			Family Practice			General Internal Medicine			
	n/N	Median proportion of healthcare visits for AGE (%)*	% missing **	n/N	Median proportion of healthcare visits for AGE (%)*	% missing**	n/N	Median proportion of healthcare visits for AGE (%)*	% missing**	p-value comparing median proportions by specialty***
Peak season	21/ 320	9	13	11/ 272	5	14	8/ 260	3	53	< .0001
Non-peak season	13/ 300	5	13	7/ 278	3	15	4/ 255	2	52	< .0001

n = median number of AGE healthcare visits each month; N = median number of all healthcare visits each month.

*% represents the median proportion of all healthcare visits that were specifically for AGE.

**% missing is for each question, specialty and season.

*** Wilcoxon test

**Table 3:** Monthly stool tests ordered among patients with AGE, by specialty and season.

	Pediatrics			Family Practice			General Internal Medicine			
	n/N	Median proportion of stool tests ordered among patients with AGE (%)*	% missing**	n/N	Median proportion of stool tests ordered among patients with AGE (%)*	% missing**	n/N	Median proportion of stool tests ordered among patients with AGE (%)*	% missing**	p-value comparing median proportions by specialty***
Peak season	2/ 21	10	12	2/11	22	13	2/8	33	52	< .0001
Non-peak season	2/ 13	12	13	2/7	29	20	2/4	33	54	< .0001

n = median number of stool tests ordered each month among patients with AGE; N = median number of all AGE healthcare visits each month.

*% represents the median proportion of stool tests ordered among patients with AGE.

**% missing is for each question, specialty and season

*** Wilcoxon test

### Availability and ordering of specific stool diagnostic tests

**Fig 1:**
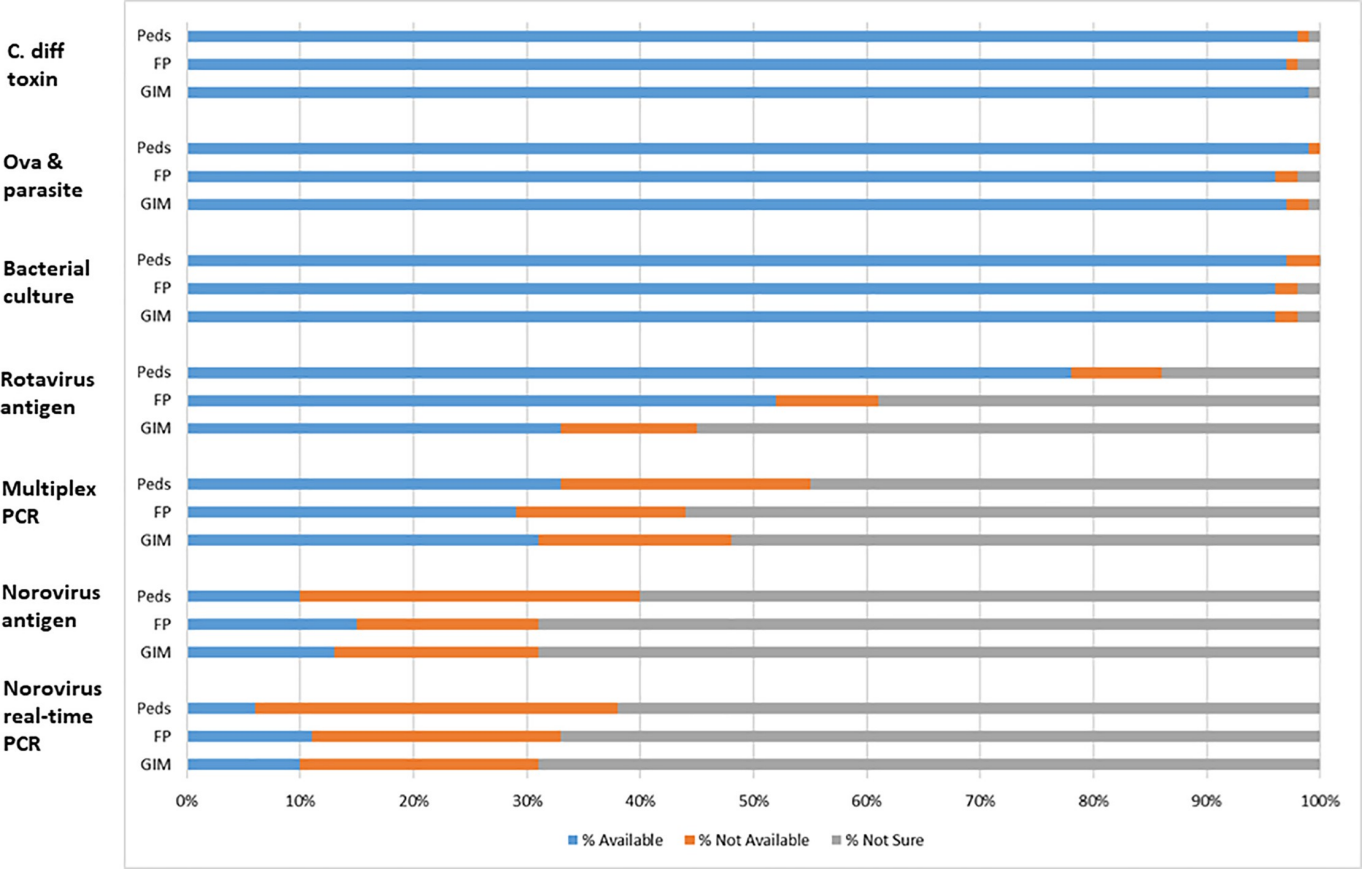
Availability of stool diagnostic tests in outpatient clinical practice, by specialty. Available refers to the proportion of providers who said they had each stool diagnostic test available for testing when managing patients with AGE in the outpatient setting. Possible responses were Available, Not available, and Not sure. GIM = General Internal Medicine; FP = Family Practice; Peds = Pediatricians.

**Fig 2:**
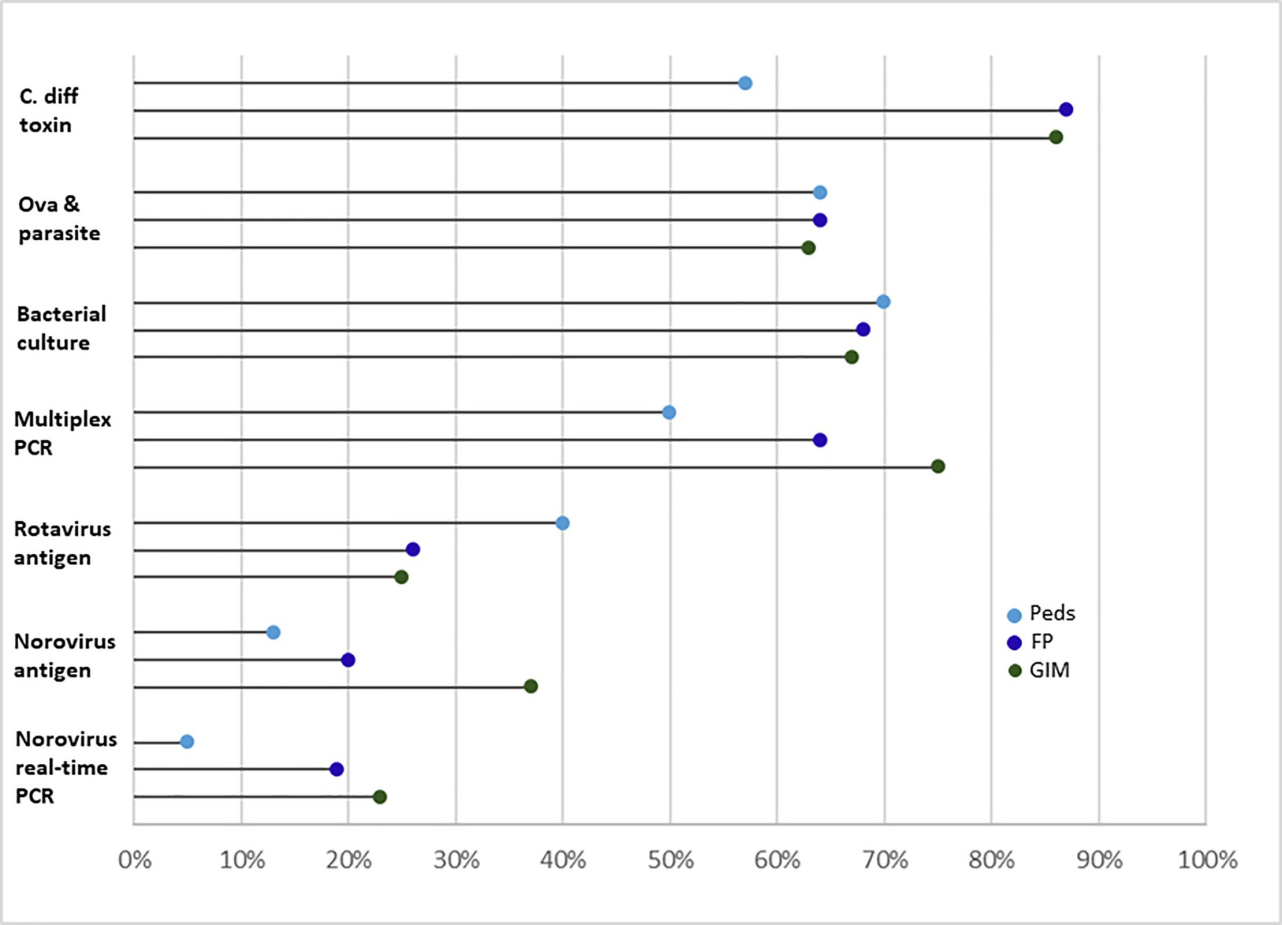
Stool tests ordered by specialty among providers for whom test is reported to be available. “Ordered” refers to the proportion of providers who reported a specific stool diagnostic test was available and that they ordered the test always, often or sometimes when managing patients with AGE in the outpatient setting. GIM = General Internal Medicine; FP = Family Practice; Peds = Pediatricians.

For all three specialties, stool tests were more often known to be available for ova and parasites (O&P), *Clostridioides difficile* (*C*. *difficile)*, and bacterial culture (>95% for all specialties) in contrast to norovirus-specific tests that included multiplex PCR, norovirus antigen, and norovirus PCR only (6–33% for all specialties) (Fig 1). Providers in all 3 specialties reported the most uncertainty about availability of the norovirus-specific tests (checked ‘not sure’ if available for 45–69% of tests) compared with other, non-norovirus tests (checked ‘not sure’ if available for <3% of tests). Among physicians reporting a given test was available, few physicians of any specialty reported ordering norovirus PCR (5%-23%) or norovirus antigen (13%-37%) tests (Fig 2).

### Factors associated with physician ordering stool diagnostic testing

**Fig 3:**
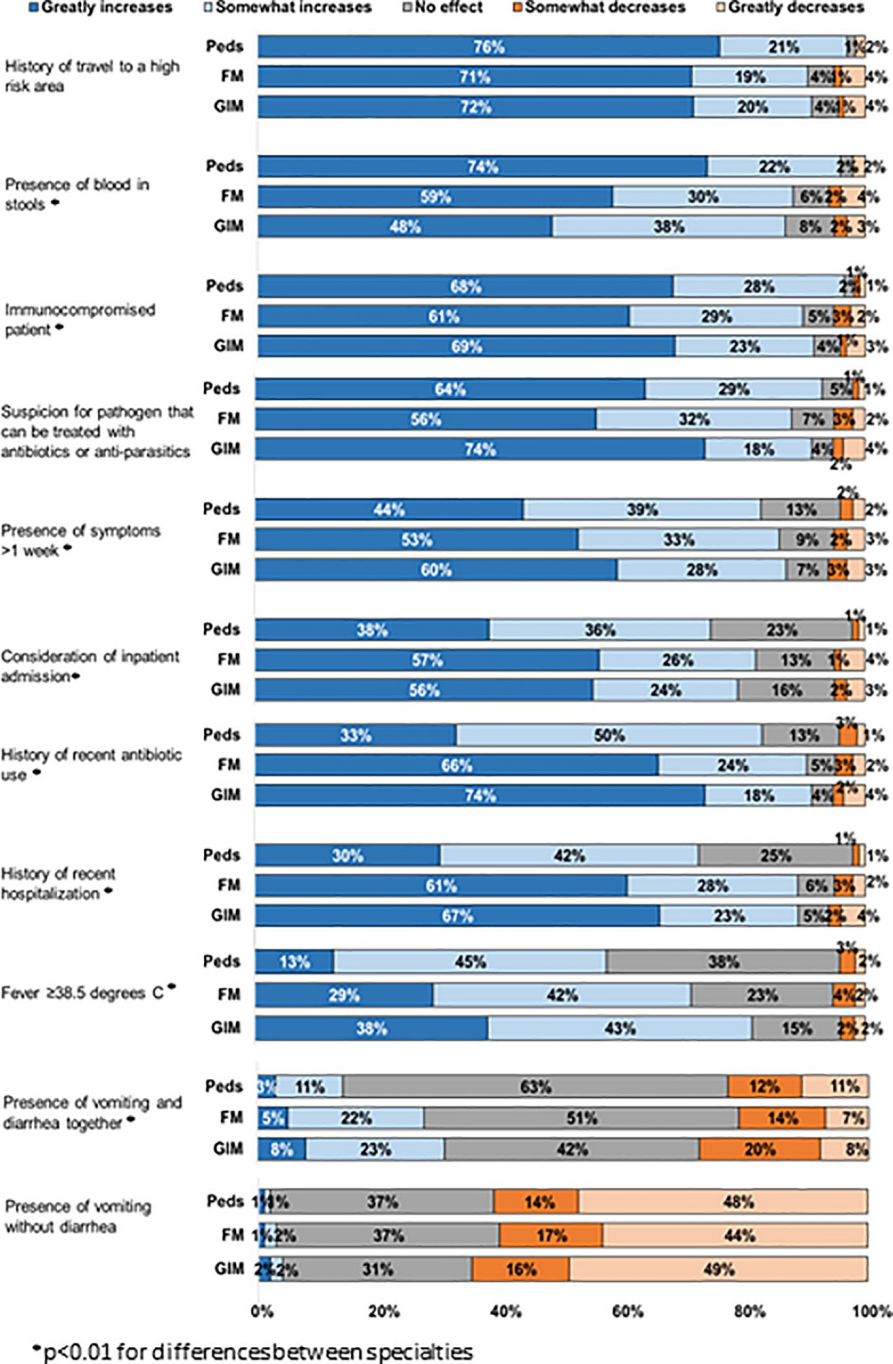
Factors associated with likelihood of stool diagnostic testing for AGE, by specialty. Other factors that were asked about that did not greatly increase stool diagnostic ordering included patient age, signs of moderate to severe dehydration, presence of mucous in stools, severe abdominal cramping, abdominal tenderness on exam, and when an outbreak of AGE is occurring. Data available in S1 Fig.

Factors most often reported as greatly increasing the likelihood of testing with minimal differences between specialties included patient history of travel to a high risk area (71–76%), immunocompromised patient (61–69%), and clinical suspicion of a pathogen that can be treated with antibiotics or antiparasitics (56–65%) (Fig 3 and S1 Fig). Peds more often reported likelihood of testing if there was blood in stool than other specialties (Peds 74%, FP 59%, GIM 49%, p<0.01). In contrast to Peds, FP and GIM more often reported greatly increased likelihood of testing if there was a history of recent antibiotic use (Peds 33%, FP 66%, GIM 74%, p<0.01), history of recent hospitalization (Peds 30%, FP 61%, GIM 67%, p<0.01), consideration of inpatient admission (Peds 38%, FP 57%, GIM 56%, p<0.01), or fever ≥38.5 C (Peds 13%, FP 29%, GIM 38%, p<0.01). For all three specialties, factors most often reported as greatly decreasing the likelihood of testing included presence of vomiting without diarrhea (44–49%) and presence of vomiting and diarrhea together (7–11%).

### Perceptions of norovirus diagnostic testing

Most providers agreed (strongly or somewhat) that norovirus testing “is helpful, because if it is positive, it helps rule out bacterial or other pathogens as causes” (57–59%). However, the majority of providers agreed norovirus testing is “usually not indicated because the results won’t change my management of the patient” (79–90%) and “is usually not indicated because it is a self-limited illness” (77–86%).

### Physician knowledge of norovirus

Most providers answered questions on norovirus treatment, immunity, transmission, and environmental persistence correctly (76–96%) (Table 4). Although two-thirds of providers knew that “After the diarrhea and vomiting from norovirus infection resolve, patients are no longer contagious” was incorrect, approximately one-third of providers answered incorrectly or did not know the answer (30–38%). Most providers were unaware that norovirus is the leading cause of AGE across all age groups (80–89%) and that alcohol-based hand sanitizers are ineffective against norovirus (51–66%).

**Table 4:** Physician knowledge of norovirus, by specialty.

Knowledge category	Statement (correct answer)	Specialty	Provider response (%)		
			Correct	Incorrect	Don’t Know
Treatment	Other than supportive care, there are no specific drugs for the treatment of norovirus (TRUE)	Peds	**96**	1	4
		GIM	**90**	0	10
		FP	**92**	0	8
Immunity	An individual can have repeated norovirus infections (TRUE)	Peds	**92**	1	7
		GIM	**87**	1	12
		FP	**87**	0	13
Transmission	Norovirus is typically spread person-to-person through fecal-oral transmission (TRUE)	Peds	**92**	5	3
		GIM	**85**	8	8
		FP	**85**	8	7
Environmental persistence	Norovirus can persist on surfaces for days and requires thorough cleaning and disinfection (TRUE)	Peds	**83**	6	11
		GIM	**83**	3	14
		FP	**76**	6	18
Shedding and infectiousness	After the diarrhea and vomiting from norovirus infection resolve, patients are no longer contagious (FALSE)	Peds	**70**	15	14
		GIM	**66**	13	21
		FP	**62**	11	26
Burden	Norovirus is the second most common cause of AGE across all age groups in the United States after rotavirus (FALSE)	Peds	**20**	61	19
		GIM	**11**	59	30
		FP	**14**	59	27
Prevention	Alcohol-based hand sanitizer is an effective method for removing norovirus from hands (FALSE)	Peds	**49**	32	18
		GIM	**38**	40	22
		FP	**34**	45	20

GIM = General Internal Medicine

FP = Family Practice

Peds = Pediatricians

### Potential barriers to future norovirus vaccines

Concerns cited as major barriers to implementing a future norovirus vaccine included if the vaccine is not covered by insurance (64–74%) and lack of adequate reimbursement for vaccination (43–50%). Factors that providers believed were ‘not at all a barrier’ or ‘minor barrier’ to new vaccine introduction included belief that “my patients won’t need this vaccine” (78–90%) and “my patients already get too many vaccines” (89–95%).

## Discussion

Our national survey of primary care Peds, FP, and GIM physicians found that only 15% of outpatients with AGE have stool tests ordered during peak AGE season, and diagnostic practices are largely focused on testing for bacteria and parasites and among high-risk patients. Viral pathogens, including norovirus, are much less likely to be identified with current diagnostic testing practices, so traditional surveillance methods that rely on clinician-ordered diagnostic testing will largely underestimate the burden of disease. Additionally, our survey found that most physicians were unaware that norovirus is a leading cause of AGE across all age groups, and that hand sanitizers are ineffective as a prevention tool for norovirus. Closing these knowledge gaps could help support norovirus prevention efforts, including potential introduction of norovirus vaccines.

Our finding that few patients with AGE have stool diagnostic testing aligns with a previous study[15], and has important implications for understanding clinician testing practices. FoodNet population-based telephone surveys from 2000–2007 found that only 15% of patients with AGE in the community seek medical attention, and of those, diagnostic testing is requested for only 13% [15]. This low stool specimen testing rate reflects clinical decision making in the face of variable patient presentations. In our survey, physicians were more likely to order testing when the patient had a higher risk presentation (e.g., travel history, blood in stool, immunocompromised). Physician specialty was also an important factor; compared with Peds, FP and GIM were more likely to test patients with fever, patients who were sick enough to consider inpatient admission or had a recent hospitalization, and those with history of recent antibiotic use. These factors can be associated with sicker patients, including those with *C*. *difficile* infections[16], bringing *C*. *difficile* to the forefront of a differential diagnosis in an adult patient with AGE with relevant symptoms and risk factors. Peds may be less likely to include bacterial pathogens high on the differential for outpatients as viruses cause most AGE and outpatient AGE visits in children [3].

These differences in clinical diagnostic testing decisions by patient and specialty are intricately tied to treatment decisions, as the severity of illness as well as the ability to treat illness may lead physicians to order tests. Indeed, all three specialties were more likely to order stool diagnostic testing if they suspected a pathogen that can be treated with antibiotics or anti-parasitics; *C*. *difficile* tests, O&P, and bacterial culture were reported as widely available. On the other hand, suspected viral or non-infectious etiology, such as patients with vomiting without diarrhea, or vomiting and diarrhea together, greatly decreased the likelihood of physician ordered stool diagnostic testing, and tests for viral pathogens were reported as less available and less likely to be ordered.

The stool diagnostic testing practices for patients with AGE identified herein have ramifications for interpreting public health surveillance of AGE pathogens and in pathogen-specific burden of disease estimates, including norovirus. If only a fraction of patients with AGE have stool specimens submitted for testing, and the tests available and ordered by clinicians are heavily weighted towards bacterial and parasitic pathogens, viral pathogens are likely to be underestimated. Additionally, the majority of providers surveyed believe norovirus testing won’t change management or is not indicated due to its self-limited nature, and did not know how norovirus testing would be helpful. From a clinical perspective, these beliefs and testing practices reflect a logical and efficient testing algorithm that minimizes the burden on the patient and the healthcare system. In mild to moderate cases of AGE, particularly in the outpatient setting where rapid point of care testing for viral pathogens is not available, testing may not be necessary or helpful for the clinician and patient management. However, from a public health surveillance standpoint, it is clear that current stool diagnostic testing practices in the outpatient setting will underestimate the burden of norovirus disease. While multi-pathogen diagnostic panels are increasingly used in inpatient and research settings [17, 18], the incidence of norovirus in the outpatient setting is approximately one order of magnitude higher than for inpatients[1], underscoring the importance of obtaining accurate estimates of norovirus disease in this patient population. Therefore, although our results do not indicate a need for changes in current physician practices, they support using robust surveillance methods to estimate norovirus burden, including prospective, population-based surveillance of laboratory-confirmed disease, as have been employed in outpatient, emergency department, and inpatient settings [3, 19, 20], to fully appreciate the disease burden of norovirus gastroenteritis. When active prospective evaluations are not possible, AGE burden studies utilizing only International Classification of Disease (ICD)-codes or passive surveillance methods may consider using this survey’s findings to weight estimates and place findings into context.

Lack of testing for all possible etiologic pathogens in all patients who present with AGE may also contribute to the physician knowledge gaps about norovirus identified herein. Interestingly, all physician specialties in this survey had knowledge gaps on norovirus burden. The high percentage of providers unaware of the large burden of norovirus disease in the United States could be due in part to the length of time most survey participants have been in practice, as compared to the relatively recent introduction of the rotavirus vaccine in 2006, resulting in norovirus surpassing rotavirus as the leading cause of medically-attended AGE in young children[3]. Furthermore, the role of norovirus among cases of AGE has been recognized relatively recently, with the advent and use in research of sensitive and specific RT-PCR assays [1]. The shift in pathogen frequencies, together with the lack of widely available diagnostic testing for norovirus as well as vaccine candidates still several years away from implementation, are all likely contributors to this knowledge gap on norovirus burden. Even among physicians for whom norovirus diagnostic tests are available, few reported ordering these tests for outpatients with AGE, further reducing opportunities to recognize norovirus disease burden.

Similarly, the fact that most providers in all specialties did not know that alcohol-based hand sanitizers (ABHS) alone are ineffective for norovirus prevention was striking. This knowledge gap could be due to several factors including variation in hand-hygiene practices among healthcare workers[21], clinical guidelines for handwashing outside of those specific for norovirus, and debates in the literature on this topic. The current CDC recommendation for norovirus prevention is to wash hands with soap and water thoroughly, with or without the use of ABHS as an adjunct in between proper handwashings [22]. This recommendation is based on the small infectious dose of virus that causes disease[23, 24], incomplete inactivation or reduction in norovirus titers or surrogates following the use of ABHS[25–28], and greater effectiveness of handwashing as compared with use of ABHS[29–32]. However, there may still be a role for ABHS in norovirus prevention and control; a randomized controlled trial in an elementary school evaluating ABHS together with surface disinfection compared to usual hand-washing and cleaning practices found a reduction in absenteeism due to gastrointestinal illness and surface detection of norovirus in the intervention arm following implementation[33]. Additionally, although CDC guidelines recommend handwashing during norovirus outbreaks, they state a preference for ABHS during routine practice if the health care worker’s hands are free of any visible soil[21]. These nuanced recommendations, together with the ubiquitous presence of ABHS in clinical practice settings in the United States[34], may partly explain the knowledge gaps identified in this survey and underscore the need for additional physician education on hand hygiene recommendations.

Our study is also notable for the lack of barriers identified by all physician groups for future norovirus vaccine introduction. Those potential barriers that were identified, including lack of reimbursement or insurance coverage, were no different from a previous study examining adult provider perceptions of barriers to vaccines[35]. With the increasing complexity and number of vaccines in both child and adult routine immunization schedules, concerns have been raised that patients may be overwhelmed by the number of shots for themselves and their children. Our survey results indicate that providers support vaccine introduction and do not believe their patients receive too many vaccines. With norovirus vaccine candidates currently in phase I and II clinical trials[5], this support will facilitate new vaccine introduction in future years.

This study has limitations. First, the response rate was lower for GIM and FP than for Peds, and some questions on testing and disease burden had high rates of missing for GIM, which limits the generalizability of our data for adult providers. With this exception, this was a nationally representative survey with membership from three major physician organizations, so responses would be expected to generally reflect perceptions of the members within each organization. Second, responses may be subject to recall bias and reflect reported practice; actual practice was not observed. Nonetheless, the responses are in line with studies utilizing other sources and methods. Finally, we did not survey patients or parents regarding future norovirus vaccine introduction; their perception of an additional vaccine may differ from that among providers. Conducting this type of survey among both physicians and patients immediately prior to or after the addition of a norovirus vaccine to routine immunization schedules would provide additional information that will help with successful implementation of a new vaccine.

In conclusion, this nationally representative survey of primary care pediatricians, family medicine and general internal medicine physicians provides new insights on several fronts for norovirus. First, the knowledge gaps on norovirus burden and prevention might be addressed through partnerships with major physician organizations to improve education for both trainees and physicians in practice. Second, the diagnostic practices for patients with AGE should continue to be monitored in light of the increasing availability of multi-pathogen diagnostic panels, and can inform ongoing surveillance to more accurately measure burden of disease. Finally, as norovirus vaccines continue to move through clinical trials, continued implementation of robust active, prospective population-based surveillance will set the stage for successful future introduction of norovirus vaccines.

## Supporting information

S1 Data Underlying dataset for analyses. (XLSX)

S1 Fig Additional factors associated with likelihood of stool diagnostic testing for acute gastroenteritis, by specialty. Peds = Pediatricians; FP = Family Practice; GIM = General Internal Medicine. *p<0.01 for differences between specialties.
(PDF)
