# Supplementary material for: Primary care physician knowledge, attitudes, and diagnostic testing practices for norovirus and acute gastroenteritis
Source: PLoS One. 2020 Jan 14;15(1):e0227890. doi: 10.1371/journal.pone.0227890 (PMC6959576; doi:10.1371/journal.pone.0227890)

S1 Fig. Additional factors associated with likelihood of stool diagnostic testing for acute gastroenteritis, by specialty.

*p<0.01 for differences between specialties

Peds=Pediatricians; FM=Family Medicine; GIM=General Internal Medicine

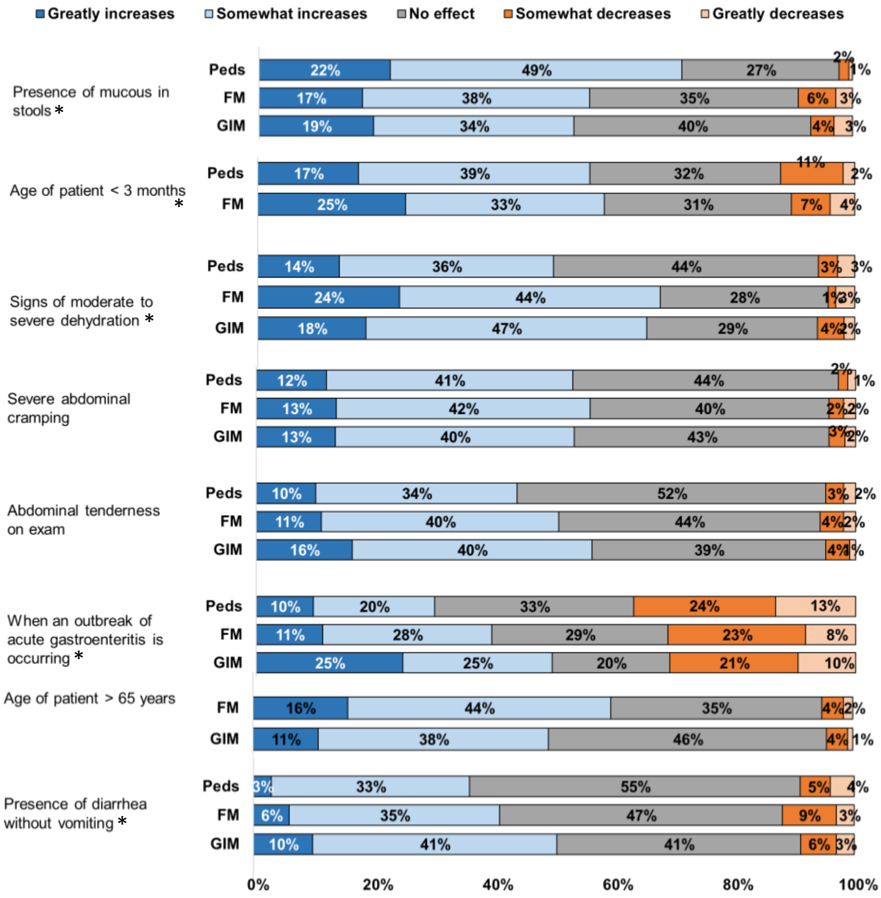

Supplement: S1 Fig — Peds = Pediatricians; FP = Family Practice; GIM = General Internal Medicine. *p<0.01 for differences between specialties. (PDF) [file pone.0227890.s002.pdf]
